# RWD insights into the use, safety and effectiveness of novel treatments

**DOI:** 10.1002/alz.084543

**Published:** 2025-01-09

**Authors:** Atsushi Iwata

**Affiliations:** ^1^ Tokyo Metropolitan Institute for Geriatrics and Gerontology, Tokyo Japan

## Abstract

In Japan, the regulatory authority approved the drug in September 2023, and on December 20, it became available for prescription country‐wide under the health insurance system. However, there are strict patient, physician, and facility requirements for the prescription of Lecanemab, and various problems are anticipated in its future implementation and widespread use in society.

Lecanemab is the first anti‐Aβ antibody in Japan, and even dementia specialists do not have sufficient knowledge and experience in its introduction, evaluation of efficacy, and evaluation and handling of side effects. In particular, it is important to establish a smooth flow of referrals from primary care physicians to specialized medical institutions and the sharing of correct awareness of the drug in order to expand the range of this drug’s use.

In addition, the Japanese government has instructed Eisai to conduct a post‐marketing surveillance of all patients in order to properly evaluate the efficacy and adverse effects of the drug. Therefore, various data will be obtained within this framework, but since some ADL scales and raw imaging data will not be collected, a registry is planned to be started by academia to supplement Eisai’s database. As a pilot study, we have established an electronic data collection system (EDC) and a mechanism for registering data to an imaging database, and named it REGI‐ALZ (Real‐world Evidence Generating Initiative for ALZheimer’s isease). In Japan, APOE genetic testing can only be done for research purposes because there is no approved in vitro diagnostic test for APOE. Therefore, RFEGI‐ALZ was designed to allow data collection for the purpose of measuring APOE. We hope that this system will serve as a reference for the registry of academia that will be established in the future.

